# AM-DMC-AMPS Multi-Functionalized Magnetic Nanoparticles for Efficient Purification of Complex Multiphase Water System

**DOI:** 10.1186/s11671-016-1434-5

**Published:** 2016-04-22

**Authors:** Yuru Ge, Yushu Li, Baiyi Zu, Chaoyu Zhou, Xincun Dou

**Affiliations:** Laboratory of Environmental Science and Technology, Xinjiang Technical Institute of Physics & Chemistry, Key Laboratory of Functional Materials and Devices for Special Environments, Chinese Academy of Sciences, Urumqi, 830011 China

**Keywords:** Fe_3_O_4_, Polymeric functionalization, Magnetic nanoparticles, Water purification, Oil water

## Abstract

Complex multiphase waste system purification, as one of the major challenges in many industrial fields, urgently needs an efficient one-step purification method to remove several pollutants simultaneously and efficiently. Multi-functionalized magnetic nanoparticles, Fe_3_O_4_@SiO_2_-MPS-AM-DMC-AMPS, were facilely prepared via a one-pot in situ polymerization of three different functional monomers, AM, DMC, and AMPS, on a Fe_3_O_4_@SiO_2_-MPS core-shell structure. The multi-functionalized magnetic nanoparticles (MNPs) are proven to be a highly effective purification agent for oilfield wastewater, an ideal example of industrial complex multiphase waste system containing cations, anions, and organic pollutants. Excellent overall removal efficiencies for both cations, including K^+^, Ca^2+^, Na^+^, and Mg^2+^ of 80.68 %, and anions, namely Cl^−^ and SO_4_^2−^, of 85.18 % along with oil of 97.4 % were shown. The high removal efficiencies are attributed to the effective binding of the functional groups from the selected monomers with cations, anions, and oil emulsions.

## Background

The rapid progress of nanoscience and nanotechnology has enabled researchers to engineer materials on the nanoscale and molecular level to a great extent [1]. Consequently, as part of the nanomaterial engineering, multi-functionalization of nanoparticles aiming at incorporating several functionalities into one single nanoparticle is of increasing interest and has been widely applied in a variety of scientific fields [2–8]. There are two major strategies for fabricating multi-functional nanoparticles: (1) individually attaching multiple functions to nanoparticles [9, 10] and (2) attaching multiple functions to nanoparticle with a single attachment point [11]. However, further applications of both strategies are hindered mainly by the tedious multistep synthesis and the associated difficulties in analytical quality control of each synthetic step. The lack of efficient and well-controlled synthesis approaches to multi-functionalize nanoparticles has become a bottleneck limiting the applications of multi-functionalized nanoparticles (MFNPs) in various fields. Therefore, simpler, faster, more efficient, and well-controlled approaches for preparing MFNPs are in urgent need.

As the representative of base materials [10, 12, 13], Fe_3_O_4_ magnetic nanoparticles (MNPs) have attracted a significant amount of interest during the past few decades owning to their merits, such as good stability, facile synthesis, high surface area, low toxicity, good biocompatibility, and especially unique magnetic responsivity [14]. The generally functionalized Fe_3_O_4_ MNPs are composed of a magnetic core, an outer functionalized shell, and commonly a silica shell in between not only serving as a protection layer by isolating the magnetic core from the external environment to achieve a better stability but also providing a biocompatible and easily modified surface enabling the covalent modification and functionalization. Although the functionalized Fe- or Co-based MNPs with single or multiple functionalities have already been applied in a variety of research fields [2, 15–23], there are still some areas in which Fe_3_O_4_ MNPs with multiple functionalities are more desired but barely explored compared to those with single functionality. The purification of industrial complex multiphase system is one of such areas, which generally contains multiple types of pollutants, such as cations, anions, and organic compounds. However, up to date, the study of functionalized Fe_3_O_4_ MNPs is limited to single-functionalization which can only remove one type of pollutant in the system, such as hydrocarbon oil [24, 25] and heavy metal ions [26]. Thus, multi-functionalized MNPs, which can adsorb and remove several pollutants simultaneously from the complex multiphase system, represent a promising agent for simple one-step complex multiphase system purification.

Acrylamide (AM)-based polymer, which can adsorb small organic emulsion droplets and is commonly used as a chemical flocculant in sludge wastewater treatment [27], plays an important role in adsorbing organic contents preventing fouling. Meanwhile, the ammonium group contained in methylacryloxyethyltrimethyl ammonium chloride (DMC) can bind with anions and serve as an anion adsorbing agent in the purification procedure. Furthermore, 2-acrylamido-2-methylpropanesulfonic acid (AMPS) could be a good candidate to remove cations due to the fact that its functional groups, amide, sulfonic acid, and carbonyl, can bind with heavy metal ions [28]. Therefore, if these three different monomers with various functional groups could be grafted onto the surface of MNPs via a very simple one-pot in situ free radical polymerization to form a functional polymeric shell, the as-prepared multi-functionalized MNPs would be used as a purification agent for adsorption and removal of various pollutants in a complex system. Oilfield wastewater, generally containing a significant amount of hydrocarbon oil and high salinity (cations and anions), is an ideal example of a complex multiphase system to illustrate the above multi-functionalized MNPs design. Furthermore, the fouling issue, which is caused by organic content and might occur rapidly during the desalination procedure resulting in loss of desalination efficiency, could be avoided effectively by utilizing the multi-functionalized MNPs.

Herein, we present a facile preparation of multi-functionalized MNPs for purification of complex multiphase industrial wastewater via a one-pot in situ polymerization of AM, DMC, and AMPS on the surface of modified Fe_3_O_4_@SiO_2_. As proved in the case of oilfield wastewater treatment, the multi-functionalized MNPs can remove both cations and anions, including K^+^, Ca^2+^, Na^+^, Mg^2+^, Cl^−^, and SO_4_^2−^, with an overall weight average removal efficiency of 83 % and with an excellent oil-removal efficiency of 97.4 %. To the best of our knowledge, this is the first trial on the multi-functionalized MNPs for the application of complex multiphase wastewater purification.

## Methods

### Materials

Ferric chloride hexahydrate (FeCl_3_·6H_2_O), tetraethyl orthosilicate (TEOS), ethylene glycol (EG), ammonia (25 wt%), sodium acetate (NaAc) were purchased from Sinopharm. Acrylamide (AM), methylacryloxyethyltrimethyl ammonium chloride (DMC, 80 wt%), γ-methylacryloxypropyltrimethoxysilane (MPS), and 2-acrylamido-2-methyl propane sulfonic acid (AMPS) were purchased from Aladdin. Polyethylene glycol (PEG, Mw = 2000) and ethanol were purchased from Tianjin Baishi Chemical Co., Ltd. Ammonium persulfate (APS) was purchased from Tianjin Hongyan Chemical Co., Ltd. Carbon tetrachloride (CCl_4_) was obtained from Tianjin Aoran Fine Chemical Co., Ltd. All of the chemicals purchased are in analytical grade and were used as received without further purification.

### Synthesis of Monodispersed Magnetic Fe_3_O_4_ Nanoparticles

Monodispersed magnetic Fe_3_O_4_ nanoparticles were synthesized according to the solvothermal method [29]. Typically, FeCl_3_·6H_2_O (2.7 g, 10 mmol) was dissolved in ethylene glycol (80 mL) to form a clear solution, followed by the addition of polyethylene glycol (3.0 g). The mixture was heated with vigorous stirring to 60 °C in an oil bath for 30 min before the addition of NaAc (7.2 g). The mixture was stirred vigorously at room temperature for another 30 min and then sealed in a Teflon-lined stainless-steel autoclave (100-mL capacity). The autoclave was heated to and maintained at 200 °C for 6 h and allowed to cool to room temperature. The black products were collected with the help of an external magnet, washed several times with ethanol and deionized water, and subsequently vacuum dried at 45 °C for 6 h.

### Synthesis of Fe_3_O_4_@SiO_2_ Core-Shell Nanoparticles

Magnetic Fe_3_O_4_ nanoparticles (0.4 g) were dispersed in the mixture of ethanol (200 mL) and deionized water (100 mL) and sonicated for 5 min. After the injection of 2 mL aqueous ammonia solution (25 wt%), tetraethyl orthosilicate (TEOS, 6.0 mL) was consecutively added to the mixture under continuous mechanical stirring. The reaction was allowed to proceed at room temperature for 7 h under mechanical stirring. The resulting Fe_3_O_4_@SiO_2_ nanoparticle products were washed with ethanol and deionized water to remove unreacted TEOS.

### Synthesis of Fe_3_O_4_@SiO_2_-MPS Core-Shell Nanoparticles

The surface modification of Fe_3_O_4_@SiO_2_ core-shell nanoparticles was carried out according to a reported procedure [30]. The preformed Fe_3_O_4_@SiO_2_ nanoparticles were dispersed in ethanol (80 mL) contained in a three-necked round-bottom flask and sonicated for 5 min. After the addition of ethanol solution of MPS (2 mL/40 mL), the mixture was refluxed at 78 °C for 24 h with continuous mechanical stirring under a nitrogen atmosphere. The surface modified Fe_3_O_4_@SiO_2_ core-shell nanoparticles with reactive C=C bonds were collected with the help of an external magnet, washed with ethanol for several times, and then vacuum dried at 60 °C for 8 h for the next step.

### Synthesis of Fe_3_O_4_@SiO_2_-MPS-AM-DMC-AMPS Core-Shell Nanoparticles

The core-shell-structured Fe_3_O_4_@SiO_2_-MPS-AM-DMC-AMPS nanoparticles were synthesized via a one-pot free radical polymerization. Vinyl-modified Fe_3_O_4_@SiO_2_ (0.3508 g) core-shell nanoparticles were dispersed in 50 mL deionized water in a round-bottom flask (100 mL) and sonicated to form a homogenous suspension. Excess amount of AM (3.1572 g), DMC (3.9465 g), and AMPS (1.0524 g) monomers were injected into the mixture, respectively, followed by nitrogen purging for 15 min. After the addition of APS (7.4 mg), the flask was sealed and heated with continuous stirring at 65 °C for 7 h in oil bath. Upon completion of the reaction, the product was harvested and washed several times with deionized water and ethanol by magnetic separation.

### Measurements and Characterization

The morphology of the samples was characterized by field-emission scanning electron microscope (FESEM, ZEISS SUPRA 55VP) and transmission electron microscope (JEM-2011 TEM, 200 kV). X-ray diffraction (XRD) patterns were recorded using a Bruker D8 Advance diffractometer with a copper target at 40 kV and 40 mA with 2θ value ranging from 20° to 80°. Fourier transform infrared (FT-IR) spectra were obtained with a Thermo Fisher Nicolet 6700 FT-IR spectrometer at room temperature using KBr pellets. Thermal analysis experiments were collected using a NETZSCH STA449F3TGA apparatus operated at a heating rate of 5 °C/min under nitrogen atmosphere. In order to evaluate desalination efficiency, each ion (Na^+^, K^+^, Mg^2+^, Ca^2+^, Cl^−^, SO_4_^2−^) was quantified by inductively coupled plasma optical emission spectrometry (ICP-OES, Agilent 735) and ion chromatography (IC, DIONEX ICS-5000) with TMS as an internal standard and CCl_4_ as eluent. Infrared photometer oil content analyzer was utilized to evaluate the oil-removal efficiency.

Oil-containing wastewater obtained from the 72# oil well of Karamay Oilfield was used to determine the desalination efficiency of the as-synthesized nanoparticles. The average value of concentration of contents in the oil-based wastewater sample used in this study was listed in Table 1. Typically, 25 mg of the pre-grounded nanoparticles were soaked into 80 mL oil-based wastewater at room temperature, followed by sonication for several minutes. Then, with the help of an external magnet, the nanoparticles were easily isolated and the supernatant was collected. The supernatant was extracted with CCl_4_ yielding the organic phase for oil content measurement, and the aqueous phase for ion content measurement to evaluate the desalination efficiency. The desalination efficiency can be calculated by the equation

**Table 1:** Average value of concentration of contents in oil-based wastewater samples used in this study

Ions (mg/L)							
Total	Cations				Anions		Oil (mg/L)
	Ca^2+^	K^+^	Mg^2+^	Na^+^	Cl^−^	SO_4_^2−^	
23860.7	191.3	63.6	42.2	11350.9	10304.2	1908.5	812.9

$$ R\left(\%\right)=\frac{C_{\mathrm{i}}-{C}_{\mathrm{f}}}{C_{\mathrm{i}}}\times 100\ \% $$

where *R* (%) represents the ion removal efficiency, *C*_i_ is the initial ion content, and *C*_f_ is the final ion content after desalination. Besides, the removal capacity of the particles is also defined as the mass of ions or oil removed in milligrams/gram of particles.

## Results and Discussion

**Scheme 1:**
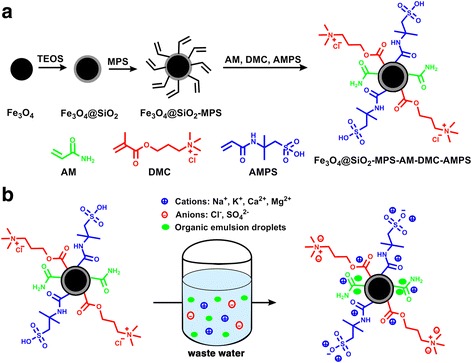
Preparation and proposed working mechanism illustration. Schematic illustration of **a** the preparation and **b** the proposed working mechanism of the Fe_3_O_4_@SiO_2_-MPS-AM-DMC-AMPS core-shell nanoparticles

The overall fabrication procedures for the Fe_3_O_4_@SiO_2_-MPS-AM-DMC-AMPS core-shell nanoparticles are schematically illustrated in Scheme 1a. First, uniform Fe_3_O_4_ nanoparticles were synthesized through a modified solvothermal reaction [29]. Second, the surface of the MNPs were coated with a thin layer of dense amorphous silica based on the hydrolysis of TEOS via the well-known Stober method [31]. The Fe_3_O_4_@SiO_2_ core-shell nanoparticles were then modified with a silane coupling agent (MPS) to form active C=C bond on the surface for the next polymerization step. Finally, the second shell of polymer was coated on the surface of Fe_3_O_4_@SiO_2_-MPS particles by a free radical polymerization of AM, DMC, and AMPS, resulting in uniform polymeric magnetic nanoparticles with core-shell structure. Due to the fact that the free radical polymerization processed randomly among the monomers, the chemical structure of the polymer could be represented as (AM)_m_-(DMC)_n_-(AMPS)_o_. The polymeric shell itself is illustrated as the black circle, while some functional side chains are shown for better understanding of the working mechanism.

The proposed complex multiphase wastewater treating mechanism of the polymeric magnetic nanoparticles was briefly illustrated in Scheme 1b. Different functional groups on the polymeric shell introduced by the three monomers were able to bind with cations, anions, and small organic emulsion droplets which are commonly found in complex multiphase wastewater. The removal of anions is realized by the ammonium chloride group in DMC via ion exchange, while the cations can be removed by not only the sulfonic acid group in AMPS via ion exchange but also the amide group on AM and AMPS via weak binding. Furthermore, as a commonly used demulsion agent, AM could also adsorb small organic emulsion droplet in the wastewater to prevent fouling, which is caused by organic content and might occur rapidly during the desalination procedure and results in loss of the desalination equipment efficiency. Therefore, the proposed polymeric magnetic nanoparticles can remove cations and anions efficiently in multiphase wastewater without fouling and easily be isolated from the system with magnetic field after purification.

**Fig. 1:**
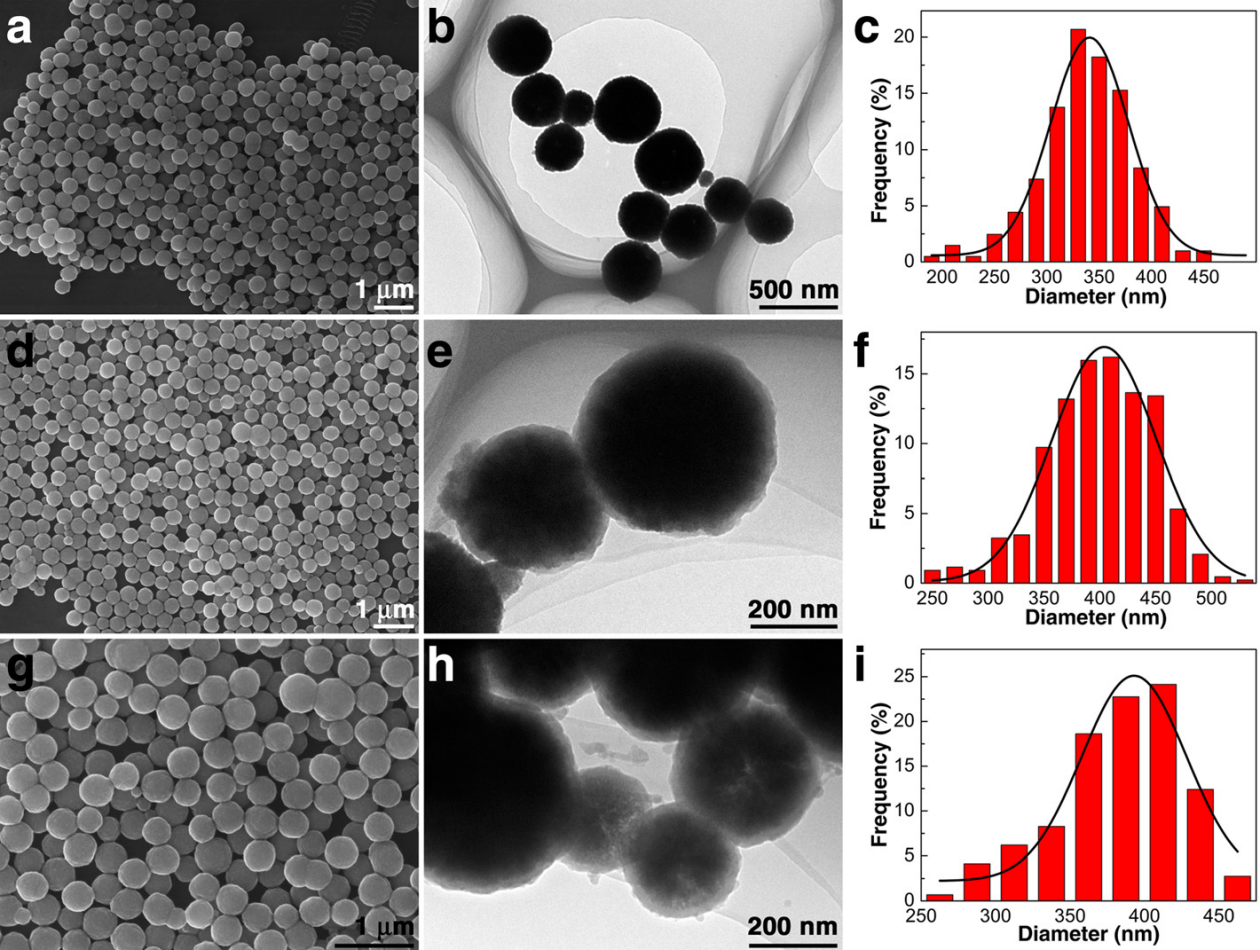
Morphological characterization of the nanoparticles along preparation. SEM images, TEM images, and size distributions of **a**–**c** magnetic Fe_3_O_4_ nanoparticles, **d**–**f** Fe_3_O_4_@SiO_2_ core-shell nanoparticles, and **g**–**i** Fe_3_O_4_@SiO_2_-MPS-AM-DMC-AMPS core-shell nanoparticles

Typical SEM and TEM images of the as-synthesized nanoparticles at each step, namely Fe_3_O_4_, Fe_3_O_4_@SiO_2_, and Fe_3_O_4_@SiO_2_-MPS-AM-DMC-AMPS nanoparticles, along with the size distributions obtained from the SEM observation, are shown in Fig. 1. Uniform Fe_3_O_4_ nanoparticles with an average diameter of 340 nm were successfully prepared (Fig. 1a–c) After coating with a thin SiO_2_ layer, the average diameter of Fe_3_O_4_@SiO_2_ increased to around 404 nm (Fig. 1d–f), corresponding to a 60 nm thick SiO_2_ layer on the Fe_3_O_4_ particles, which is able to protect the Fe_3_O_4_ core and also provide the possibility for further functionalization. In the SEM and TEM images, even in the size distribution chart of Fe_3_O_4_@SiO_2_-MPS-AM-DMC-AMPS nanoparticles (Fig. 1g–i), there is no obvious diameter increase observed. The reason for this could be addressed to that the polymeric shell formed in the present study was too thin to be observed in TEM results. Due to the fact that only functional groups exposed on the surface of the polymeric shell are effective during the purification procedure, the limited thickness of the polymeric shell would not affect the purification properties of the MNPs in a negative way. In fact, the thin polymeric shell layer is preferred in this case. Other than the primary free radical polymerization, the three monomers in excess could also react with each other and consume the functional groups. If there is only a very thin layer of polymer formed on the surface of the MNPs, the overreaction between monomers could be prevented effectively.

**Fig. 2:**
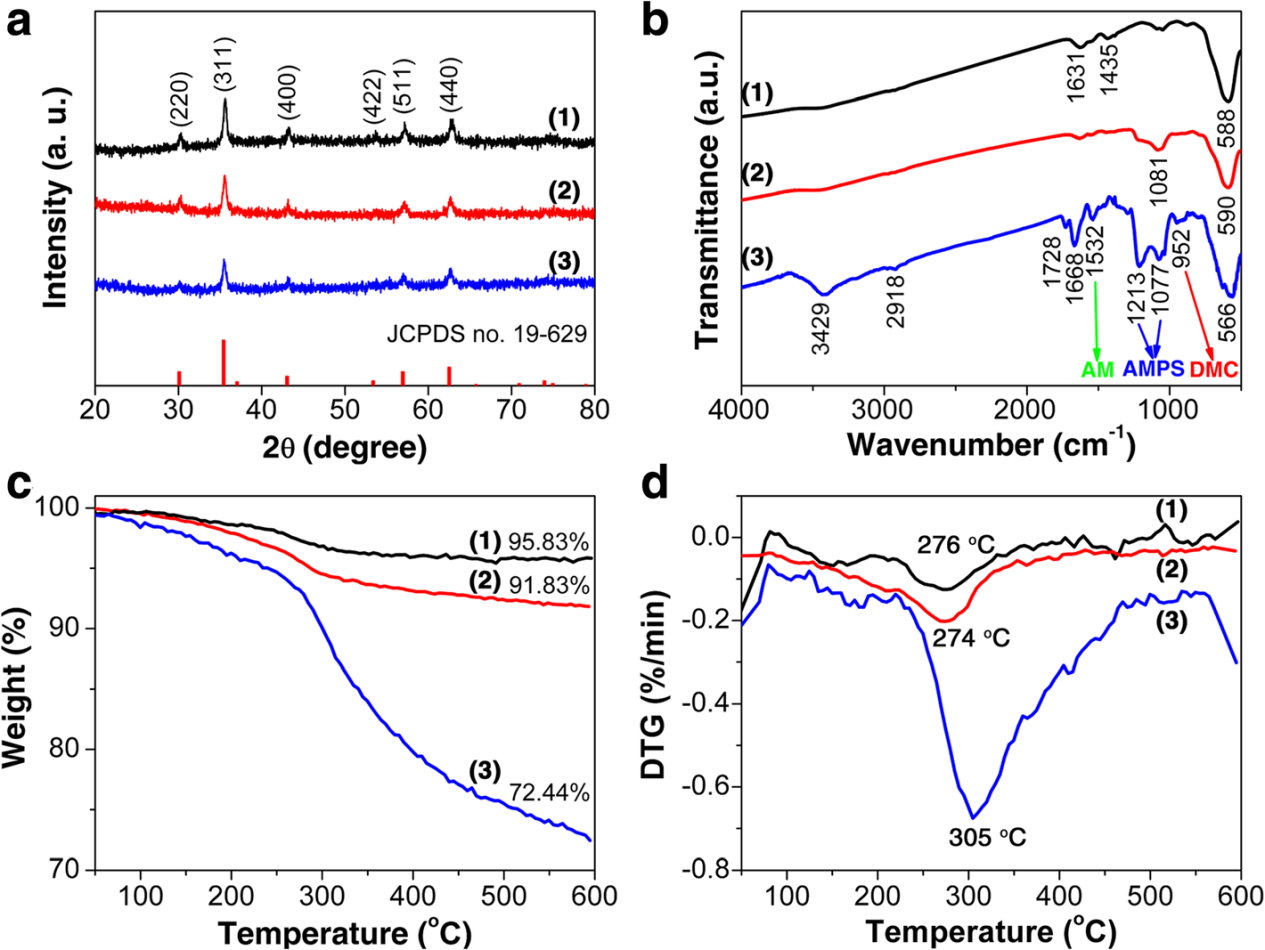
Structural and thermal characterization of the nanoparticles along preparation. **a** XRD patterns, **b** FT-IR spectra, **c** TGA, and **d** DTG curves of (*1*) Fe_3_O_4_, (*2*) Fe_3_O_4_@SiO_2_, and (*3*) Fe_3_O_4_@SiO_2_-MPS-AM-DMC-AMPS nanoparticles

In order to investigate the crystal structures of the as-prepared materials, XRD experiments were conducted. As shown by curve 1 in Fig. 2a, the diffraction peaks for Fe_3_O_4_ nanoparticles located at 2θ values of 30.2°, 35.6°, 43.3°, 53.5°, 57.2°, and 62.8° are sharp and strong and could be well indexed to the (220), (311), (400), (422), (511), and (440) planes of face-centered cubic (fcc) phase magnetite (JCPDS no. 19-629), respectively, [32] confirming the successful synthesis of the magnetite nanoparticles with good crystallinity. Besides, the broadening feature of the recorded peaks indicates that the size of the grains in Fe_3_O_4_ nanoparticles is in the nanometer scale. According to Scherrer’s relation: *D* = 0.9 *λ*/*β*cos*θ*, where *D* is the average crystalline size, *θ* is the Bragg diffraction angle, and *β* is the full width at half maximum, the average size of the Fe_3_O_4_ grains is calculated to be 19 nm using the (311) peak for Fe_3_O_4_. Similar diffraction peaks are also observed for Fe_3_O_4_@SiO_2_ and Fe_3_O_4_@SiO_2_-MPS-AM-DMC-AMPS samples, as shown by curves 2 and 3 in Fig. 2a, suggesting that the Fe_3_O_4_ nanoparticles are well retained in the magnetic composite nanoparticles [25]. Meanwhile, the amorphous structure of silica can also be confirmed since no diffraction peaks corresponding to SiO_2_ are observed.

Successful preparation of Fe_3_O_4_@SiO_2_-MPS-AM-DMC-AMPS was also confirmed by FT-IR spectroscopy (Fig. 2b). For Fe_3_O_4_ cores (curve 1 in Fig. 2b), the absorption band at around 590 cm^−1^ is assigned to Fe–O stretching [33], while the bands observed at around 1631 and 1435 cm^−1^ correspond to the carboxylate group of sodium acetate, which acts as the stabilizer for Fe_3_O_4_ nanoparticles during the preparation. The presence of sodium acetate represents that the Fe_3_O_4_ nanoparticles have excellent dispersibility in polar solvents, which is beneficial for the next SiO_2_ coating and modification processes. For Fe_3_O_4_@SiO_2_ (curve 2 in Fig. 2b), the absorption band associated to Si-O-Si vibrations appeared at 1081 cm^−1^ indicates the successful formation of silica shell on the magnetite surface. The C=C stretching band at 1644 cm^−1^ comes from MPS, which acts as the surface modify agent of Fe_3_O_4_@SiO_2_ particles. For Fe_3_O_4_@SiO_2_-MPS-AM-DMC-AMPS (curve 3 in Fig. 2b), several new absorption bands appear as evidences of the formation of organic shell on Fe_3_O_4_@SiO_2_ nanoparticles. The absorption band at 3429 cm^−1^ is assigned to be the stretching vibrations for N–H bond in amide groups consisted in AM and AMPS moieties. The characteristic absorption bands for C=O in ester groups are found at 1728 cm^−1^. The band at around 1668 cm^−1^ is associated to the C–N bond in amide group, while the deformation band of –NH_2_ in amide group is found at 1532 cm^−1^, demonstrating the existence of AM moiety. The presence of DMC is confirmed by the characteristic absorption band for quaternary ammonium group at around 952 cm^−1^. Moreover, the asymmetric and symmetric bands of SO_2_ in sulfonic acid group are at 1213 and 1077 cm^−1^, along with the band corresponding to the –OH bond in sulfonic acid at 2918 cm^−1^, indicating the appearance of AMPS moiety. However, The Si–O–Si band is covered by the symmetric band of SO_2_ in sulfonic acid group from AMPS at 1077 cm^−1^. And the Fe–O bond seems to be displaced about 20 to 566 cm^−1^ owing to the fact that the Fe–O band at about 590 cm^−1^ is covered by the vibration band of C–C backbone with side chains in the organic shell, which also fall in the region around 570 cm^−1^. Moreover, the absence of C=C stretching band (1644 cm^−1^) indicates the completion of the reaction between MPS on the surface of the SiO_2_ and the three monomers. Therefore, it can be concluded from the inferred spectral analysis that the organic shell was successfully synthesized by AM, DMC, and AMPS as raw materials.

To quantitatively determine the composite of the obtained Fe_3_O_4_@SiO_2_-MPS-AM-DMC-AMPS, thermogravimetric analysis (TGA) was conducted. Figure 2c, d shows the TGA and DTG curves of the obtained Fe_3_O_4_, Fe_3_O_4_@SiO_2_ and Fe_3_O_4_@SiO_2_-MPS-AM-DMC-AMPS, respectively. The weight loss of 4.17 wt% for Fe_3_O_4_ particles, which existed in the temperature range of 246–276 °C (curve 1 in Fig. 2d), could be attributed to the degradation of the stabilizer sodium acetate and the desorption of surface adsorbed water molecules (curve 1 in Fig. 2c). From the curve of Fe_3_O_4_@SiO_2_ (curve 2 in Fig. 2c), a weight loss of 8.17 wt% was observed at around 274 °C (curve 2 in Fig. 2d), which was believed to be related to the desorption of water molecules bonded to SiO_2_ layer. Moreover, after being grafted with MPS-AM-DMC-AMPS, the weight loss of the composite particles was increased to 27.56 wt% at about 305 °C (curve 3 in Fig. 2c, d). These results clearly imply that AM-DMC-AMPS has been successfully grafted onto the surface of Fe_3_O_4_@SiO_2_-MPS.

**Fig. 3:**
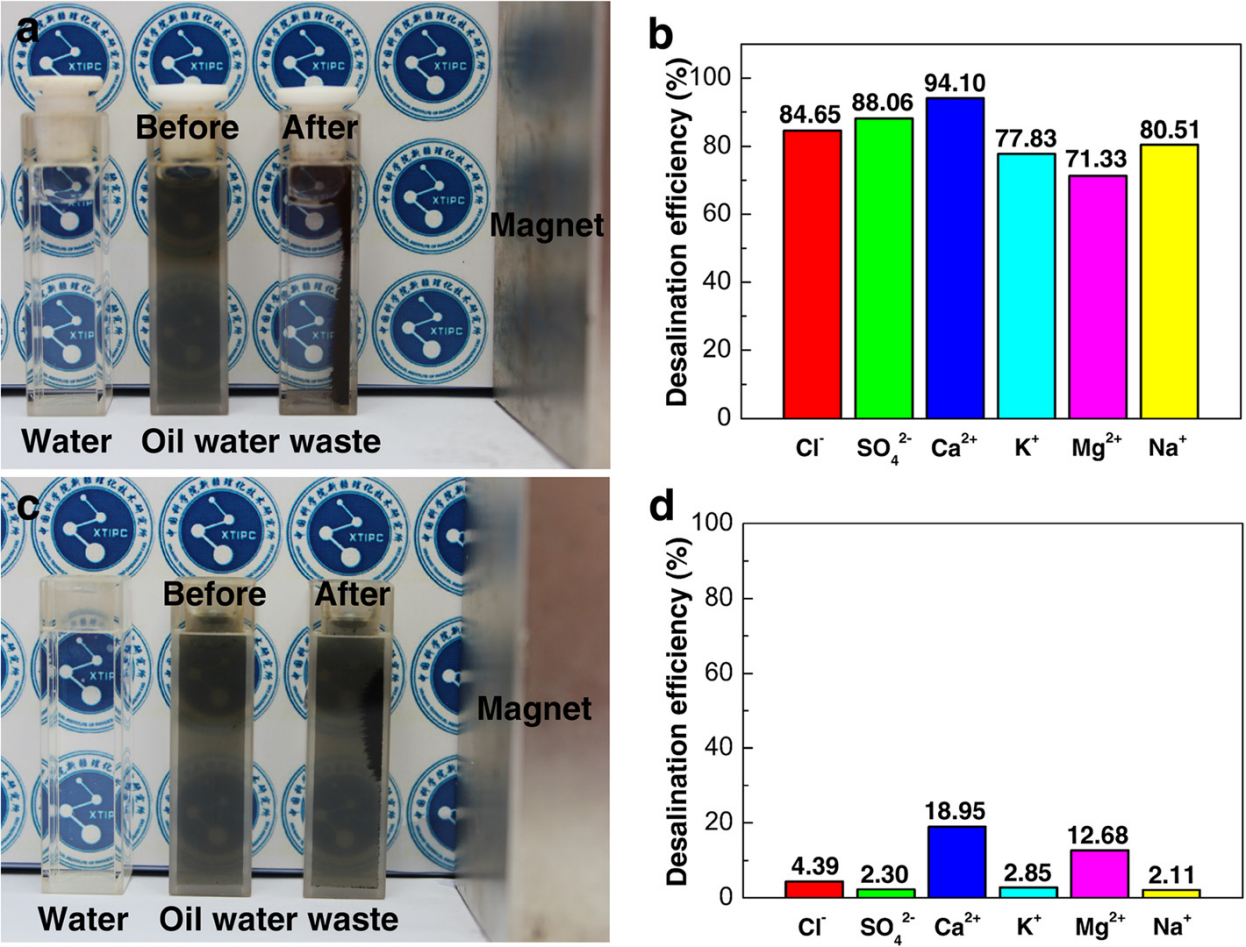
Purification performance of the nanoparticles towards oilfield wastewater. Photographs of deionized water, oilfield wastewater before and after treated with **a** Fe_3_O_4_@SiO_2_-MPS-AM-DMC-AMPS and **c** Fe_3_O_4_ under external magnet field, respectively, and the corresponding desalination efficiency of **b** Fe_3_O_4_@SiO_2_-MPS-AM-DMC-AMPS and **d** Fe_3_O_4_ for each ion

The purification property of the as-synthesized nanoparticles was evaluated by using the magnetic nanoparticles to treat the oilfield wastewater obtained from Karamay oil field as an example of complex multiphase wastewater, as shown in Fig. 3. The major ions contributing to the salinity of this oilfield wastewater sample are chloride (43.18 %), sodium (47.57 %), and sulfate (7.99 %), while calcium (0.80 %), potassium (0.27 %), and magnesium (0.18 %) are minor ions quantified by ion chromatography, as shown by Table 1. The oil content in this oilfield wastewater sample was measured to be 812.9 mg/L using inferred oil content analyzer.

The sludge oilfield wastewater became obviously much clearer after treatment associating to the removal of oil emulsions in the system while using Fe_3_O_4_@SiO_2_-MPS-AM-DMC-AMPS nanoparticles to carry out the purification (Fig. 3a), which was further proven by the IR oil content analyzer results. After treated with the nanoparticles, the oil content of the sample dropped dramatically to 20.8 mg/L, indicating a great oil-removal capacity of 2534.7 mg/g of the nanoparticles. On the other hand, the ion analysis of the supernatant further confirms the excellent desalination efficiency of the as-synthesized Fe_3_O_4_@SiO_2_-MPS-AM-DMC-AMPS nanoparticles to remove both cations and anions remarkably (Fig. 3b). It is shown that the Fe_3_O_4_@SiO_2_-MPS-AM-DMC-AMPS nanoparticles can remove most of the major ions, 84.7 % of chloride, 80.5 % of sodium, and 88 % of sulfate, with a total weighted average desalination efficiency of 83 %. The overall removal capacities of the nanoparticles for cations and anions are 30073.9 and 33289.6 mg/g, respectively. Thus, the as-synthesized Fe_3_O_4_@SiO_2_-MPS-AM-DMC-AMPS nanoparticles with oil-removal property show high desalination efficiency, providing a simple one-step purification method of complex multiphase wastewater system like oilfield wastewater. In contrast, the sludge oilfield wastewater remained visibly unchanged after treatment while utilizing Fe_3_O_4_ nanoparticles without surface modification as the purification agent (Fig. 3c). Furthermore, Fe_3_O_4_ nanoparticles shows a poor ion removal capacity with the best ion removal rate of 18.95 % for calcium (Fig. 3d). This result provides a vivid demonstration that our multi-functionalization design of MNPs with AM, DMC, and AMPS works effectively for multiphase complex system purification.

As previously discussed, the capability of desalination was introduced to the magnetic nanoparticles by the functional polymeric shell synthesized via a one-pot free radical polymerization of three different monomers. Thus, the ratio of the three monomers is a significant factor affecting the desalination efficiency of the magnetic nanoparticles. A series of Fe_3_O_4_@SiO_2_-MPS-AM-DMC-AMPS nanoparticles were synthesized with different AM: DMC: AMPS monomer ratios and applied for the purification of the identical oilfield wastewater sample to evaluate their desalination properties.

**Fig. 4:**
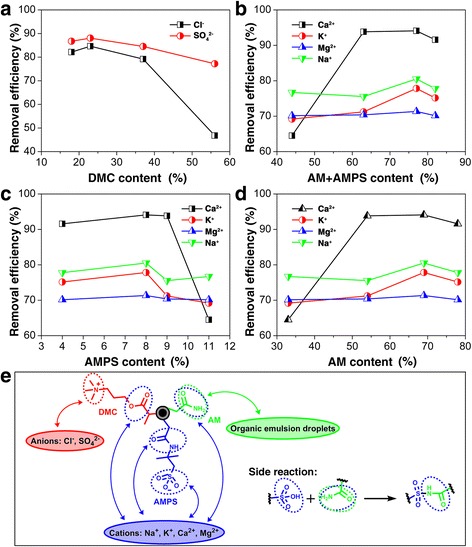
Relationship between surface functionalities and the purification performance. Ions removal efficiencies as a function of **a** DMC content, **b** AM + AMPS content, **c** AM content, **d** AMPS content and **e** the proposed binding interaction between functional groups and pollutants

The removal efficiencies of anions, i.e., Cl^−^ and SO_4_^2−^, as a function of the DMC content are plotted in Fig. 4a. It is obvious that with the increase of DMC content, the removal efficiencies for both Cl^−^ and SO_4_^2−^ anions increase first and then decrease. The removal efficiency dropped much more significantly for Cl^−^ with increasing the content of DMC in the polymeric shell, which can be attributed to the introduction of chloride ions into the system by the amino chloride group on DMC. Besides, the removal efficiency of SO_4_^2−^ decreases slightly with further increase of DMC due to the fact that the active adsorbent sites for Cl^−^ and SO_4_^2−^ anions got decreased. Therefore, with the increase of the amount of DMC, there is a balance between the increase of the adsorbent sites for Cl^−^ and SO_4_^2−^ anions and the increase of the introduced chloride anions. Meanwhile, both AM and AMPS have the amide groups which can bind with cations, i.e., Na^+^, K^+^, Mg^2+^ and Ca^2+^. Therefore, the removal efficiencies of cations as a function of the AM + AMPS content are plotted in Fig. 4b. It is shown that with the increase of the AM + AMPS content, the removal efficiencies of all the four cations all increased firstly and then kept stable, indicating the achievement of ion exchange equilibrium. With a further incensement of AMPS + AM content, the cations removal efficiencies started to drop. One possible explanation for this phenomenon is that with the increase of the amount of both AMPS and AM monomers, there are side reactions consuming functional groups, such as sulfonic acid and amino groups. When considering AMPS content only, the removal efficiency changing trend is different (Fig. 4c). With the increase of the amount of AMPS, the cation removal efficiencies increased slightly first and then dropped dramatically, which could also be attributed to the side reactions during the polymerization process. Although both AM and AMPS have a binding effect with cations, AMPS with the sulfonic acid group acts as the main component for cation adsorption. Thus, the consumption of sulfonic acid group affects the cation removal efficiencies greatly. The changing trends of the removal efficiencies for the cations with the increase of the content of AM are similar to those of AMPS + AM, as shown in Fig. 4d. To vividly explain the reason for the desalination performance, the binding interaction between the functional groups on the polymeric shell with ions and organic emulsion droplets is further illustrated in Fig. 4e. One also can find that the side reaction during the polymerization would affect the ratio of the functional groups. Thus, it is demonstrated that the functional groups on the three monomers, namely DMC, AM, and AMPS, are able to bind with anions, organic droplets, and cations, respectively. Furthermore, the designed Fe_3_O_4_@SiO_2_-MPS-AM-DMC-AMPS nanoparticles with an optimized monomer ratio of AM: DMC: AMPS (3:3:1) is a suitable purification agent for complex multiphase system.

## Conclusions

In summary, to realize the purification of complex multiphase industrial wastewater, a facile one-pot free radical polymerization of AM, DMC, and AMPS was designed to form a polymeric shell on the surface of MPS-functionalized Fe_3_O_4_@SiO_2_ nanoparticles. To evaluate the overall removal efficiency, the as-synthesized Fe_3_O_4_@SiO_2_-MPS-AM-DMC-AMPS nanoparticles were applied as the purification agent for oilfield wastewater, a great example of complex multiphase system. Excellent removal efficiencies for cations (80.68 %) and anions (85.18 %) along with oil (97.4 %) were achieved with the AM: DMC: AMPS ratio of 3:3:1. The design concept illustrated here presents a facile multi-functionalization method of magnetic nanoparticles which can fulfill specific application demands.
